# Fragmentation and integration in long-term care for older adults living with dementia in mainland China: a polycentric governance framework synthesis

**DOI:** 10.3389/fpubh.2026.1873910

**Published:** 2026-10-30

**Authors:** Hong Sun, Xinnan Yu, Haoyu Zheng, Min Lai, Zijun Zhu

**Affiliations:** 1 College of Urban Construction, Zhejiang Shuren University, Hangzhou, China; 2 Ninth Architectural Design Institute, Zhejiang Provincial Institute of Architecture Design and Research, Hangzhou, China; 3 College of Architecture and Art, Hebei University of Architecture, Zhangjiakou, China; 4 Hebei Green Building Collaborative Innovation Center, Hebei University of Architecture, Zhangjiakou, China

**Keywords:** care fragmentation, dementia, integrated care, long-term care, mainland China, meta-governance, polycentric governance

## Abstract

**Objectives:**

To examine how long-term care for older adults living with dementia is organised across family, community, institutional, and cross-level settings in mainland China; identify where fragmentation occurs; and examine mechanisms relevant to integration.

**Methods:**

We conducted a systematic review with a theory-informed framework synthesis. CNKI and the Web of Science Core Collection were searched for empirical studies published from 2006 to 2026. Eligible reports were independently screened, appraised with design-appropriate tools, and synthesised across care domains, with polycentric governance used as a sensitising framework.

**Results:**

The review included 340 reports representing 321 underlying studies or projects. Within individual care domains, families carried most day-to-day care and coordination responsibilities, community services had uneven capacity for sustained support and linkage, and institutional expansion was not consistently matched by dementia-specific staffing, competence, or organisational support. Directly documented fragmentation concerned referral and transition, information continuity, and the transfer or sharing of responsibility, while financing and eligibility interfaces were less directly studied. Directly evaluated interventions were concentrated in caregiver support and selected setting-based programmes, while more direct integration evidence concerned continued cross-setting support, defined coordination roles, referral and feedback, and multidisciplinary or community-linked collaboration. System-level integration was seldom directly evaluated.

**Conclusion:**

Long-term dementia care in mainland China comprises multiple service components, but their connections remain uneven. The findings identify cross-setting coordination, information continuity, follow-up and clearer responsibility as priorities for development and evaluation, while financing and eligibility require further investigation.

## 1 Introduction

Dementia progressively affects cognition, behaviour, and everyday functioning. Its consequences extend well beyond diagnosis and episodic treatment, creating continuing needs for personal care, behavioural and psychological support, rehabilitation, social participation, decision support, caregiver assistance, and end-of-life care (1–4). This review focuses on long-term care for older adults living with dementia in mainland China, specifically the continuing health, personal, and social support provided across family, community, and institutional settings. It does not address general long-term care for other populations with dependency. Because such care involves coordination among multiple actors and settings, its organisation is also a governance issue.

China faces a large and growing dementia burden. An estimated 17 million people were living with dementia in 2021, and demand for continuing care is expected to rise as the population ages (5, 6). Care is commonly described across three domains. Families remain the main source of support; community care includes relevant primary-care, public-health, and social-care functions such as identification, follow-up, caregiver support, and referral; and institutions provide residential or higher-intensity care when needs can no longer be met at home (7). These domains describe points along a care pathway, not uniform categories of providers. Their coexistence does not in itself mean that care is coordinated.

Most research has examined one care domain at a time. Studies of family-based care have focused on caregiver capacity, burden, coping, home adaptation, and unmet support needs (8–13). Studies of community-based care have considered identification, access, caregiver support, collaboration, and workforce capacity (14–17). Studies of institutional care have addressed specialised environments, person-centred practice, service quality, staff competence, and organisational models (18–23). Taken together, this literature provides substantial evidence on care within particular settings, but less is known about the cross-setting connections involving referral and follow-up, information transfer, financing, and changes in responsibility between formal and informal care (4, 24).

In this review, fragmentation refers to weak or broken connections across the care pathway rather than service limitations within a single setting alone (25). Capacity deficits, poor quality, unmet need, inequitable access, caregiver burden, and knowledge deficits may shape care experiences but do not by themselves constitute fragmentation. Integration, in turn, concerns the alignment of actors and functions needed to maintain continuity across settings (26). Existing studies suggest that families often take on coordination work without comparable formal support, while stigma, affordability, and uneven access to formal support can complicate care-seeking and continuity (27–30). Care components may therefore coexist without forming a coherent pathway.

Polycentric governance offers a useful way to examine this problem because dementia care involves several decision-making centres that are separate but interdependent (31, 32). The framework distinguishes governance actors from care settings and directs attention to how responsibilities, authority, and resources are distributed among these interdependent centres. Multiple centres can support local adaptation and draw on different forms of expertise, but weak coordination can disperse responsibility and undermine continuity (31, 33). Meta-governance complements this perspective by conceptualising public stewardship as the setting of common rules and support for coordination without absorbing the roles of families, providers, or community organisations (33, 34).

The review addressed three questions: how long-term care is organised across family, community, institutional, and cross-level settings in mainland China; where and why fragmentation occurs; and which governance or implementation mechanisms may improve integration. By synthesising evidence from both care domains and their connections, the review uses polycentric governance, complemented by a meta-governance perspective on public stewardship, to examine how the organisation of responsibilities and coordination shapes fragmentation and integration across the care system.

## 2 Methods

### 2.1 Review design and reporting

The included literature comprised qualitative, quantitative, mixed-methods, observational, intervention, programme-evaluation, and empirical case-study designs, making statistical pooling inappropriate. We therefore conducted a systematic review with a theory-informed framework synthesis, drawing on best-fit framework synthesis and the framework method (35, 36). Reporting followed PRISMA 2020 (37, 38). The review was not prospectively registered, and no formal written protocol was established before the review commenced.

The review question was structured using PICo, with the operational eligibility criteria specified in Section 2.2. Polycentric governance was used as a sensitising perspective rather than as a fixed causal model (31, 32). It directed attention to whether assigned responsibilities were matched by decision-making authority and resources, how care domains were linked, and where accountability lay, while allowing themes from the studies to modify the initial framework. The review treated underlying studies or projects, rather than individual reports, as the unit of interest (39, 40).

### 2.2 Eligibility criteria

Eligibility was assessed against the following population, phenomenon, context, evidence-type, and relevance criteria.

#### 2.2.1 Inclusion criteria

1. Population: Older adults living with dementia in mainland China, or caregivers, professionals, organisations, and public actors directly involved in their long-term care. Dementia had to be clinically documented, identified using a validated method, or explicitly defined by the study. No fixed age threshold was imposed, but participants had to be described as older adults.
2. Phenomenon of interest: Empirical findings on the provision, use, organisation, coordination, financing, workforce, quality, continuity, accessibility, integration, or governance of sustained dementia care and support.
3. Context: Family care, community care, institutional care, or transitions and interfaces among these domains in mainland China.
4. Evidence type: Original qualitative, quantitative, mixed-methods, observational, intervention, programme-evaluation, or empirical case studies published in Chinese or English.
5. Relevance: Mixed-population studies were included only when dementia-specific findings were separately identifiable.

#### 2.2.2 Exclusion criteria

We excluded studies conducted solely outside mainland China; reviews, commentaries, protocols, duplicate reports without unique empirical findings, and conference records without unique data; and non-empirical reports. Studies were also excluded when they focused on diagnosis, medication, clinical technology, psychometric validation, or disease mechanisms without a substantive long-term-care component; when the population did not include older adults living with dementia or relevant care actors; when findings concerned only mild cognitive impairment, general cognitive decline, or unverified symptoms; or when they did not address the review question. Supplementary Table S1 lists the included reports and their linkage to the same underlying study or project where applicable.

### 2.3 Information sources and search strategy

We searched the China National Knowledge Infrastructure (CNKI) and the Web of Science Core Collection for Chinese- and English-language records published between 1 January 2006 and 30 June 2026. The final CNKI search was run on 6 July 2026 and the Web of Science search on 7 July 2026. CNKI was chosen for its coverage of Chinese-language research on care practice and implementation in mainland China, while Web of Science added multidisciplinary English-language coverage. Supplementary Table S2 provides the database fields, limits, filters, search dates, and full strategies.

The search combined terms for dementia, Alzheimer disease, cognitive impairment, and their Chinese equivalents with terms covering care settings and functions, caregivers, continuity, referral, coordination, financing, long-term-care insurance, medical-social integration, and governance. Broad cognitive-impairment terms were used to improve sensitivity, but inclusion required dementia-specific evidence relevant to long-term care. We also checked references and companion reports linked to included studies. A supplementary reference-list sensitivity check of recent English-language reviews with broader international and Chinese database coverage was conducted to examine search completeness (Supplementary Table S3).

### 2.4 Critical appraisal

Methodological quality was appraised with the relevant Joanna Briggs Institute checklist for each study design and with the Mixed Methods Appraisal Tool for mixed-methods studies (41, 42). We did not calculate a common score or use quality as an automatic exclusion threshold. Instead, concerns about design, sampling, data collection, analysis, and inference affected the weight given to individual findings. Findings with important design limitations, including major confounding, absence of an appropriate comparator for effectiveness claims, substantial attrition, or serious measurement or reporting concerns, were restricted to cautious descriptive or supporting use. Appraisal judgements and their implications for synthesis are reported in Supplementary Tables S4, S5.

### 2.5 Study selection and full-text eligibility assessment

Two reviewers independently screened titles and abstracts and then independently assessed the full texts of all retrievable reports retained after screening using the same eligibility criteria. Disagreements at either stage were resolved through discussion. Four reports could not be retrieved and were recorded separately as reports not retrieved rather than as full-text exclusions. The complete study-selection process is presented in Figure 1.

**Figure 1:**
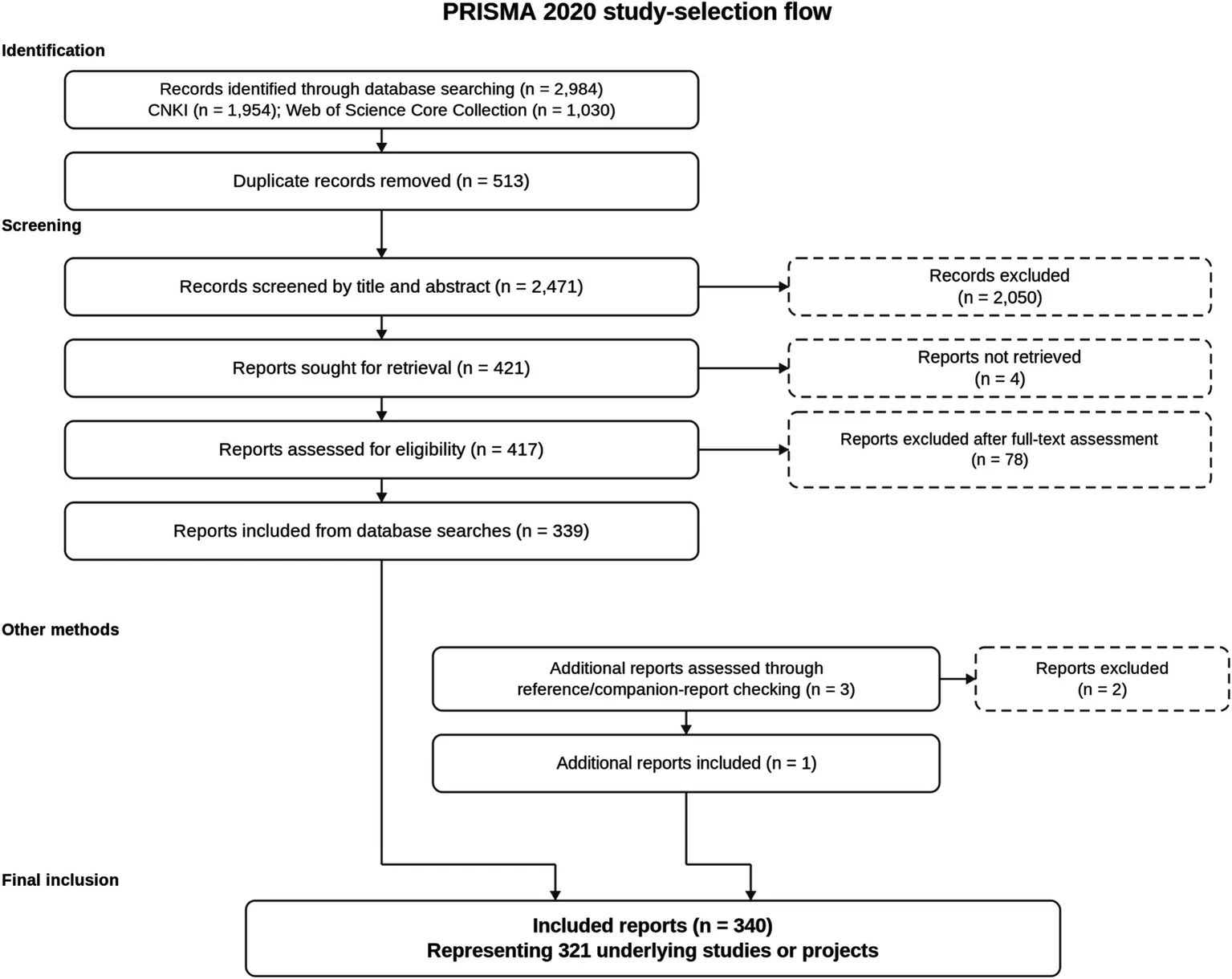
PRISMA 2020 flow diagram of study identification, screening, full-text eligibility assessment, and inclusion. The review included 340 reports representing 321 underlying studies or projects.

### 2.6 Data extraction, actor classification, and framework synthesis

Study characteristics and findings were extracted using a standardised form. Reports arising from the same underlying study or project were linked to avoid double counting while retaining distinct report-level findings. Companion reports were retained when they added distinct findings, but overlapping samples were not counted as independent evidence.

Actor, care-domain, ownership, and functional codes were kept separate. Actors were grouped as people living with dementia; informal support actors, mainly family caregivers and other unpaid networks; formal service providers; and public actors responsible for stewardship, financing, or accountability. Formal providers were further distinguished as healthcare, social-care, or organised civil-society providers. Ownership was recorded as an organisational attribute rather than as an actor category, and studies could contain more than one actor role. Actor categories were therefore non-mutually exclusive.

Findings were organised across family care, community care, institutional care, and cross-level interfaces. The first three were treated as care domains. Cross-level interfaces captured the relationships and coordination processes linking them. Community and institutional care were therefore coded as settings rather than as governance actors.

The initial categories came from polycentric governance and were revised in response to themes found in the evidence (35, 36). Primary-study findings were first coded descriptively and then mapped to four governance dimensions: responsibility and decision-making authority, capacity and resources, links among actors and settings, and accountability. Fragmentation was coded as an explicit break or weak connection across actors, services, responsibilities, information, financing or eligibility, or referral and transition processes. Evidence provenance was classified as directly evaluated mechanisms, observational or qualitative support, participant- or primary-author recommendations, and review-author governance inferences. Because the findings were heterogeneous, they were synthesised narratively. Policy and contextual sources informed the Introduction and Discussion but were not used as evidence in the Results. Detailed coding rules, appraisal weight, and evidence provenance are provided in Supplementary Tables S4, S5.

## 3 Results

The 340 included reports represented 321 underlying studies or projects. The findings are organised at three related levels: limitations within family, community, and institutional care; fragmentation at cross-setting interfaces; and evidence relevant to integration.

### 3.1 Study selection and evidence profile

Of the 2,471 records screened by title and abstract, 2,050 were excluded and 421 reports were sought for full-text retrieval. Four reports could not be retrieved, leaving 417 reports assessed for eligibility; 78 were excluded and 339 were included. Three additional reports were assessed through reference or companion-report checking, of which one was included and two were excluded. The final evidence base therefore comprised 340 reports representing 321 underlying studies or projects (Figure 1).

The 80 full-text exclusions comprised studies conducted outside mainland China (*n* = 26), studies without dementia-specific evidence (*n* = 3), reports without a substantive long-term-care component (*n* = 12), studies not involving older adults (*n* = 3), reviews or other non-empirical reports (*n* = 33), and mixed-population studies in which dementia-specific findings could not be separately identified (*n* = 3). Full exclusion reasons are listed in Supplementary Table S6.

The 340 reports were published between 2006 and 2026 and covered family, community, institutional, and cross-level care. Table 1 summarises the evidence base, and Supplementary Table S1 gives report-level details. Cross-sectional and observational studies constituted the largest part of the evidence base, alongside a substantial body of intervention and quasi-experimental studies. These heterogeneous designs were useful for examining context and relationships but provided limited support for causal inference or quantitative comparison.

**Table 1:** Profile of the included evidence.

Characteristic	Summary
Included evidence	340 reports representing 321 underlying studies or projects
Publication period	2006–2026
Sources	CNKI (*n* = 293); Web of Science (*n* = 46); reference/companion checking (*n* = 1)
Care domain	Family care; community care; institutional care; cross-level interfaces
Design profile	Cross-sectional/observational/methodological (*n* = 187); qualitative (*n* = 41); intervention/quasi-experimental (*n* = 91); longitudinal/cohort (*n* = 8); case/practice/service evaluation (*n* = 5); mixed methods (*n* = 8)
Actor categories*	People living with dementia and/or informal support actors (*n* = 308); formal healthcare providers (*n* = 119); formal social-care providers (*n* = 35); organised community or civil-society support providers (*n* = 22); public stewardship, financing, and accountability actors (*n* = 16)
Geographical profile	Evidence predominantly derived from studies conducted in individual urban locations and provincial-capital settings; limited national or multi-regional coverage and limited evidence from rural settings
Methodological appraisal	Used to calibrate interpretive weight rather than as an exclusion threshold

*Actor categories were non-mutually exclusive because individual reports could involve more than one actor role.

Studies of people living with dementia and informal caregivers were much more common than studies of formal social-care, financing, stewardship, or accountability actors. Direct contributions from people living with dementia were identified in only nine of the 340 included reports, with most evidence concerning this group derived from caregiver or proxy reports and clinical measures. Evidence examining referral, information continuity, financing, or the transfer of responsibility across settings remained less extensive than evidence concerning care within individual settings.

Evidence was also geographically concentrated. Most studies were conducted in individual cities, institutions, communities, hospitals, or provincial systems, particularly in eastern China and provincial capitals. Evidence from national or multi-regional samples and rural settings was limited. Estimates of prevalence, service use, and pilot performance from individual settings were therefore treated as setting-specific rather than nationally representative.

### 3.2 Family care: responsibility without commensurate support

Family care was the dominant arrangement and often included service navigation and coordination as well as day-to-day support. A multicentre clinical survey conducted across 30 provincial regions found that family members cared for 91% of people with Alzheimer’s disease (43). Families managed personal care, behavioural symptoms, safety decisions, service contacts, information transfer, and decisions about institutional placement. Filial obligation, cost, trust, and concern about moral judgement could delay or discourage institutional care (44, 45). In practice, many families acted both as care providers and as the main coordinators of care.

Caregiver outcomes varied, but high levels of strain were common. In Wuxi, 55% of family caregivers of people living at home screened positive for psychological problems (46). In Shanghai, the mean Caregiver Burden Inventory score was 65.9, indicating substantial burden across several domains (9). Longitudinal studies found different trajectories: burden was stable for some caregivers but rose slowly or quickly for others, while family resilience either declined or improved over time (47, 48). These patterns show why average scores may obscure families at greatest risk and why assessment may need to be repeated. Affiliate stigma, lack of respite, and limited diagnostic, professional, and financial support, especially in rural areas, were also linked to greater burden (29, 45). Dyadic qualitative studies further showed that emotional interaction and stigma were experienced by both people living with dementia and their family caregivers (49, 50).

Formal services rarely matched the breadth of family responsibility. Caregivers reported low expectations of assistance and asked for respite, financial help, professional advice, and dependable community services (44). Home-care studies described limited dementia knowledge and little continuing guidance after contact with hospitals (51). Qualitative work also found that families managed complex medical, nursing, rehabilitation, and daily-care needs without effective primary-care support or specialist outreach (12). Some made restraint and safety decisions without clear professional, legal, or policy guidance (52). The evidence points to a persistent gap between what families were expected to do and the resources, guidance, and formal support available to them.

### 3.3 Community care: an incomplete intermediary layer

Community and primary-care services were expected to identify needs, support caregivers, maintain follow-up, and connect home care with specialist and institutional services. These functions were not performed consistently. In Beijing’s Yuetan community, community institutions first identified 17% of cases and only 20% of patients had access to dementia medication within the community; assessment, referral, follow-up, caregiver instruction, and counselling were also limited (53). In Hunan Province, 76% of respondents reported a need for community dementia care, but only 42% of communities had standardised management and community nurses’ awareness of comprehensive prevention and care measures was 57% (54). These figures describe gaps between demand and capacity in the studied settings, not national coverage.

Studies of the primary-care workforce reported heavy workloads, competing responsibilities, limited dementia-specific training, and insufficient time among primary-care workers and general practitioners (55, 56). Community nurses also had gaps in dementia knowledge, caregiver guidance, and awareness of available community resources (57). Screening, education, home visits, and caregiver support were documented in some study settings, but often as short-term projects rather than routine services backed by stable funding, referral procedures, and case-management authority.

The existence of a programme did not necessarily indicate that community services had the capacity to deliver sustained support. In one evaluation, a family-physician-team-guided programme improved learning behaviour but did not reduce caregiver burden or improve quality of life or social support. Barriers included the absence of dementia support from essential primary-care services, weak financing and performance incentives, caregivers’ limited time or digital access, and difficulty balancing standardisation with individual needs (58). Other community-based interventions showed more favourable outcomes when ongoing support, home-based contact, and professional guidance were combined, including improvements in caregiver burden, social support, and dementia-care knowledge (59). Community services therefore occupied an intermediary position in the care structure, with uneven capacity affecting both support within the community domain and their ability to connect families with other parts of the care pathway.

### 3.4 Institutional care: expansion without consistent dementia-specific capability

Institutional care included residential long-term-care facilities, nursing homes, and dedicated dementia-care units. Provision had grown, but general availability did not always amount to dementia-specific capacity. In a survey of 412 institutions across 15 provinces and municipalities, 76% admitted people with dementia and 44% had dedicated dementia-care units (60). Larger institutions tended to have stronger specialist capacity, while shortages of professional services and care workers remained common (60). Bed supply and specialised provision could not therefore be treated as equivalent.

Knowledge, person-centred practice, and organisational support differed markedly between institutions. Care workers often had limited knowledge of dementia and were less likely to use person-centred approaches (61). In Hunan dementia units, organisational support was the lowest-scoring component of person-centred care (62). In grassroots institutions, the registered-bed-to-assistant ratio was 17.9:1 and the current-resident-to-assistant ratio was 10.3:1; competence also varied with shift patterns, job satisfaction, engagement, and attitudes towards older people (63). More recent multicentre evidence reinforced these workforce constraints, showing that heavier workloads and limited dementia training were associated with greater caregiver burden, while stronger caring ability was associated with lower burden (64). Together, these findings indicate that institutional expansion was not consistently matched by adequate staffing, dementia-specific training, or workforce capability.

Studies conducted in individual institutional settings reported safety concerns and behavioural complexity that could not be addressed by increasing bed numbers alone. Abuse risk or abusive tendencies were associated with behavioural symptoms, functional dependence, caregiver fatigue, burden, and training gaps (65, 66). Another study found that 95% of residents with dementia had shown at least one behavioural or psychological symptom in the previous month; family contact, environmental control, functional status, and dementia severity were included in the reported model (67). These findings were specific to their study settings and were not treated as national prevalence estimates.

Practice reports and interventions conducted in individual institutions described dedicated units, environmental modification, workforce development, and multidisciplinary care; some intervention studies reported improvements in selected care outcomes (68, 69). Most of this evidence, however, came from single organisations or short follow-up periods. It did not establish whether the models could be sustained or transferred at scale. The main issue was therefore not simply whether institutions existed, but whether expansion was accompanied by appropriate staffing, specialist knowledge, person-centred practice, and effective quality oversight.

### 3.5 Cross-setting interfaces and fragmentation

#### 3.5.1 Referral and transition

Moves between hospital, home, community, and institutional care were often organised by families rather than planned as part of a care pathway. Ethnographic work showed that institutional placement commonly followed marked deterioration or crisis and changed, rather than removed, the family’s burden, adding financial, moral, and relational pressures (70). Home-care studies similarly described complex needs without reliable specialist outreach, rehabilitation guidance, or primary-care support (12).

Collaborative models linking hospitals, community services, and families provided examples of structured cross-setting care. Hospital-community-family management and three-level management involving hospital specialists, community doctors, and caregivers were associated with improvements in selected patient and caregiver outcomes after discharge or during community follow-up (71, 72). A community-based programme combining digital support, home visits, and teleconsultation also reduced caregiver burden and improved social support and dementia-care knowledge (59), reinforcing the value of sustained professional contact across community and home settings. Across these models, explicit follow-up roles and participation across settings were recurring components of care delivery, although their routine implementation at scale was not established.

#### 3.5.2 Information continuity

Problems with information transfer were reported separately from other forms of discontinuity. Among patients with Alzheimer’s disease attending different levels of medical institutions, overall continuity-of-care experience was low, and information continuity was the lowest-scoring dimension (73). Routine discharge advice and telephone contact also appeared inadequate. One WeChat-based continuing-care programme reported better caregiver knowledge, patient activities of daily living, and safety outcomes than routine guidance (74).

Qualitative studies described inconsistent exchange of information among hospitals, emergency services, community teams, nursing homes, and families (75). One multidisciplinary pilot model for homebound care assigned a team manager and specified referral, feedback, and cross-professional processes, implying that these functions were not reliably part of routine care (76). The problem extended beyond clinical records to needs assessments, care plans, caregiver observations, and clarity about who had taken over responsibility.

#### 3.5.3 Financing and eligibility

Direct evidence on financing was less common than evidence on caregiver burden or workforce capacity. Families reported substantial out-of-pocket costs and lost work or income and asked for financial support and respite (44, 45). In one social-support programme, limited effects were linked to the exclusion of caregiver support from essential primary-care packages and to weak incentives for providers (58).

A provincial performance evaluation reported improvement in several indicators of access, affordability, and management, alongside continuing or worsening constraints in bed supply, caregiver availability, resource allocation, and people-centred integration (23). Across the evidence base, financing studies concentrated mainly on household financial pressure, affordability, gaps in funded support, and provider incentives; studies directly examining financing or eligibility arrangements across care settings were uncommon.

#### 3.5.4 Responsibility and accountability

Several studies described unclear or weakly connected responsibilities across actors and settings. A field study in Taiyuan described poorly connected formal and informal networks, gaps in authority, unequal resources, and uneven professional capacity across hospitals, communities, institutions, and families (75). A case study of a publicly built, privately operated institution also showed that mixed ownership did not by itself produce specialised dementia care when responsibilities, standards, workforce development, and supervision were unclear (22).

Similar mismatches occurred within care relationships. Institutional care workers described moral distress when family-led decisions conflicted with professional judgement or when staffing, knowledge, and organisational support prevented them from providing the care they considered appropriate (77). At home, families made decisions about restraint and safety without clear professional or legal guidance (52). Across cross-level studies, responsibility gaps were most visible when care was transferred or shared without clear authority, information, resources, or coordination arrangements.

### 3.6 Evidence relevant to integration

Directly evaluated interventions were concentrated in three areas: support for family caregivers, continued support across community and home settings, and dementia-specific care within institutions. For family caregivers, personalised leisure activities, mindfulness-based stress reduction, sustained problem-focused community nursing, collaborative nursing, and telephone-based behavioural activation were associated with improvements in selected outcomes including caregiver burden, distress, sleep, depressive symptoms, and positive caregiving experiences (78–82). Across community and home settings, interventions that combined continued follow-up with defined professional support reported improvements in selected patient and caregiver outcomes, including activities of daily living, medication adherence, caregiver self-efficacy, burden, social support, and dementia-care knowledge (59, 71, 72). In institutional settings, caregiver training and multidisciplinary care were associated with improvements in selected hygiene, functional, and quality-of-life outcomes (69, 83).

Intervention effects were not uniform across studies. A non-randomised self-management programme improved self-efficacy, social support, and symptom-management processes but did not show a clear between-group difference in total behavioural and psychological symptoms (84). A two-year community nursing study reported improvement, but unclear randomisation and other limitations prevented a confirmatory interpretation (85). Another evaluation found greater engagement without improvement in caregiver burden, quality of life, or social support when financing, incentives, digital access, and personalisation remained poorly aligned (58).

Other reports described dedicated dementia-care areas, environmental adaptation, locally developed standards, medical-nursing-rehabilitation links, and staff development. These practices had been implemented, but the available evidence was mainly descriptive and did not support comparative claims about effectiveness (68). Caregiver feedback on a comprehensive intervention suggested benefits from peer contact and positive caregiving experiences, while also identifying financial and emotional pressure, inconvenient delivery, and a preference for community, home-based, or digital formats (86).

Needs reported by participants were similar across care domains. Families asked for respite, financial help, home visits, professional guidance, counselling, peer support, and accessible community services (12, 44, 86). Mental-health providers called for tiered and coordinated training for formal providers, community workers, and informal caregivers, covering dementia knowledge, diagnosis, communication, counselling, safety, restorative care, stress management, and practical skills (17). Other studies identified a need for standardised assessment, explicit referral and feedback, shared care information, and continued follow-up (53, 73, 76).

Financial assistance, long-term-care funding, public standards, monitoring, and stewardship were frequently recommended or included in descriptive models, but were rarely implemented and evaluated. Directly evaluated interventions were concentrated in caregiver support and selected setting-based programmes. More direct integration evidence concerned continued cross-setting support, defined coordination roles, referral and feedback, and multidisciplinary or community-linked collaboration. System-level arrangements involving financing, shared information infrastructure, or accountability were seldom directly evaluated.

### 3.7 Framework synthesis

The framework synthesis brought these three levels of evidence together. Family, community, and institutional care showed distinct constraints in support, capacity, and dementia-specific capability. Reports directly examining interfaces described weaknesses in connections among actors and settings and mechanisms intended to maintain continuity. Among the 365 coded evidence units, 12 explicitly addressed fragmentation or linkage failures and 36 described integration mechanisms or coordinated models. Together, these directly related units accounted for 48 units (13.2%) of the coded corpus. Table 2 summarises the empirically observed actor–function mismatches and distinguishes empirically supported mechanisms relevant to integration from governance-informed propositions derived from the synthesis.

**Table 2:** Actor–function mismatches and integration implications across care domains and cross-level interfaces.

Care domain and principal actors	Expected functions	Empirically observed actor–function mismatches	Empirically supported mechanisms relevant to integration	Governance-informed integration propositions
Family care: People living with dementia; informal support actors	Daily care, safety, behavioural support, service navigation, information transfer, and participation in placement decisions	Families frequently undertook extensive direct care and service coordination without commensurate professional guidance, respite, financial support, or clear formal authority. Caregiver burden, psychological distress, delayed service use, stigma, and crisis-driven decisions were repeatedly reported (12, 29, 43–52).	Caregiver-focused interventions reported improvements in selected outcomes through personalised activities, mindfulness-based support, sustained problem-focused nursing, collaborative nursing, behavioural activation, home-based professional support, and continued follow-up (78–82, 86).	Formal recognition of the family coordination role; routine access to caregiver assessment, respite, guidance, and funded support; clearer interfaces between informal care and formal services.
Community care: Formal healthcare, social-care, and organised civil-society providers; public stewardship actors	Early identification, assessment, caregiver support, routine follow-up, case management, referral, and linkage to specialist and institutional services	Dementia-specific knowledge, time, funding, incentives, case-management authority, referral procedures, and information exchange varied markedly across settings. Some programmes remained project-based rather than embedded in routine service structures (53–58).	Continued follow-up, home-based contact, defined professional support, hospital–community–family collaboration, teleconsultation, and community nursing were associated with improvements in selected patient and caregiver outcomes (58, 59, 71, 72).	Stable commissioning and funding for continuing community dementia support; explicit coordination responsibility; standardised assessment and referral processes; routine rather than project-based linkage across home, primary care, and specialist services.
Institutional care: Formal healthcare and social-care providers; informal support actors; public stewardship actors	Specialised and higher-intensity care; behavioural support; safety; person-centred practice; planned admission and family involvement	Expansion in institutional provision was not consistently accompanied by adequate staffing, dementia-specific competence, organisational support, person-centred practice, or quality oversight. Workforce burden and training limitations were recurrent (60–69).	Dementia-specific staff education, dedicated care arrangements, environmental adaptation, and multidisciplinary care were associated with improvements in selected care processes and outcomes, although much of the evidence was single-site or short term (68, 69, 83).	Minimum dementia-specific workforce and training expectations; organisational support for person-centred care; stronger quality oversight; clearer links between institutions, families, community services, and external clinical providers.
Cross-level interfaces: People living with dementia; informal support actors; formal service providers; public stewardship actors	Planned referral and transition, shared information, aligned financing and eligibility, continuous coordination, and explicit accountability	The clearest breaks occurred when responsibility crossed settings without dependable referral, feedback, information transfer, funding alignment, or clearly assigned authority. Families often organised transitions themselves; direct evidence on financing and eligibility interfaces remained comparatively limited (22, 23, 52, 58, 70–77).	Hospital–community–family models, multidisciplinary coordination, continued post-discharge follow-up, defined referral and feedback processes, and sustained professional contact across settings provided direct empirical examples of cross-setting integration (59, 71, 72, 74, 76).	Closed-loop referral architecture; transferable shared-care information; explicit coordination responsibility; aligned financing and eligibility rules; clearly assigned accountability across organisations.

Empirically supported mechanisms refer to interventions or care arrangements evaluated in primary studies. Governance-informed propositions are synthesis-level implications derived from the observed evidence and the polycentric governance framework and should not be interpreted as directly evaluated system-level interventions.

## 4 Discussion

### 4.1 Principal findings and contribution of the review

Earlier studies have largely described unmet needs within individual care settings. By examining family, community, institutional, and cross-level care together, this review distinguishes three related levels of evidence: constraints within individual care domains, fragmentation at the interfaces between them, and mechanisms relevant to integration. Within family, community, and institutional care, the evidence showed substantial limitations in formal support, sustained community capacity, and dementia-specific institutional capability. Reports directly examining interfaces identified continuity problems involving referral and transition, information transfer, and the sharing or transfer of responsibility (22, 23, 43, 53, 60, 70–77).

The cross-setting synthesis also provides an analytical contribution. Across the evidence base, families often absorbed coordination responsibilities that were not reliably assigned elsewhere in the care pathway (9, 12, 29, 44–48, 51–58). Admission to an institution may alter family responsibility without ending it (70). A service can therefore have substantial internal capacity while the wider pathway still fails at referral, information transfer, handover of responsibility, or follow-up. The distribution of evaluated evidence also shows that direct evaluation was more developed for interventions within particular care domains than for system-level integration across them (58, 59, 69, 71, 72, 78–85). The small proportion of evidence units directly addressing fragmentation or integration indicates that existing dementia care research has more often documented service barriers and local practices than systematically examined governance interfaces. Section 4.2 examines how these empirical patterns interact across the care trajectory, whereas Section 4.3 considers their theoretical implications through polycentric governance and public stewardship.

### 4.2 Empirical interpretation: interacting drivers across the care trajectory

These different levels of constraint interact across the care trajectory. Family involvement will remain essential because dementia care is continuous, but involvement should not become a substitute for professional, financial, and coordination support. Behavioural symptoms, dependency, stigma, financial strain, filial expectations, and fear of judgement affect help-seeking, yet their effects depend on service availability, affordability, trust, and quality (8, 9, 12, 13, 27–29, 44–48, 51, 52, 87). Treating reliance on families mainly as a cultural preference risk overlooking service conditions that can be changed.

Community and primary-care teams are well placed to recognise needs, support caregivers, maintain follow-up, and guide access to other services. To do so, they need dementia-specific skills, authority to refer, access to specialist advice, reliable information systems, protected time, and recurrent funding (14–17, 53–58, 88–91). Some evaluated programmes indicate how stronger support within a care domain can also contribute to continuity across settings: continued professional contact, home-based support, and structured collaboration improved selected patient and caregiver outcomes, although routine implementation and long-term scale-up remain less well established (58, 59, 71, 72, 78–81, 83–86). Institutional capability depended on staffing, person-centred practice, behavioural support, safety, dementia-specific competence, organisational support, and effective links with outside services (18–24, 60–69, 92–98). Cross-level fragmentation is most evident when these setting-specific limitations are compounded by failed handovers, including delayed referral, disrupted information transfer, unclear responsibility, or absent follow-up (12, 23, 58, 70–76). Regional differences in fiscal capacity, workforce supply, insurance coverage, and affordability shape each of these processes. Because the evidence was derived mainly from eastern and urban settings, findings from individual programmes and institutions should not be assumed to be nationally representative (45, 99).

### 4.3 Theoretical contribution: polycentric governance and public stewardship

At the theoretical level, polycentric governance helps explain why fragmentation cannot be attributed to a single organisation or to the existence of multiple actors itself. Families, providers, community organisations, financing bodies, and public authorities have different but interdependent roles. The framework therefore shifts attention from individual organisations to the alignment of responsibilities, authority, resources, and cross-setting coordination among multiple decision-making centres (31, 33, 34). Without such alignment, responsibility is easily dispersed and families become the default coordinators of a system over which they have little authority. Integration, in turn, does not require eliminating multiple decision-making centres, but making their interdependence workable.

Public leadership in this context is better understood as meta-governance than as central control. The synthesis suggests that public authorities can support coordination by establishing common rules and accountability while preserving professional judgement, local adaptation, and co-production (31–34, 100–102). Community teams require authority to assess needs and initiate referral; hospitals need to connect clinical care with discharge planning; institutional providers need discretion within enforceable standards; and people living with dementia and their families should contribute to care planning without being left to coordinate the pathway alone. The central governance challenge is not simply to strengthen each organisation, but to make their interdependence workable across care settings.

### 4.4 Policy and implementation priorities

One governance implication of the synthesis is that coordination responsibility and a minimum set of cross-setting functions should be made explicit. Depending on local arrangements, this role could sit with an existing health, civil-affairs, eldercare, medical-social integration, or long-term-care insurance body, provided that its authority and accountability are explicit. Core functions would include service navigation, referral and escalation, regular review of care and caregiver needs, and reliable access to professional guidance and support (12, 17, 44, 53–59, 71–76, 86). The priority is reliable functional coverage, not the creation of the same new organisation everywhere.

Financing and eligibility remain less clearly defined in the current evidence base. Existing studies mainly document household financial pressure, affordability problems, gaps in funded support, and provider incentives, whereas cross-setting financing and eligibility arrangements have received much less direct examination (23, 30, 44, 45, 58). These arrangements should therefore be made visible in policy design and implementation because they shape who can access support, under what conditions, and across which stages of care.

Workforce development, information transfer, referral, and accountability should be planned together. Training can improve knowledge and selected care outcomes, but sustained improvement also depends on staffing, supervision, specialist support, and time for coordination (17, 55–58, 61–64, 69, 83, 92). Practical implementation priorities therefore include clearer referral and feedback arrangements, transferable care information, and defined responsibility at transitions between settings (71–76). Evaluation should give more weight to continuity, equity, caregiver well-being, person-centred quality, safety, workforce stability, and costs across sectors than to activity counts alone.

### 4.5 Strengths, limitations and research priorities

A major strength of this review is its cross-setting synthesis of 340 reports representing 321 underlying studies or projects across family, community, institutional, and cross-level dementia care. The synthesis brings together evidence on setting-specific constraints, cross-setting discontinuities, and mechanisms relevant to integration, while using the polycentric-governance framework to interpret how authority, resources, coordination, and accountability shape these patterns. Linking companion reports, applying design-appropriate appraisal, and distinguishing different forms of evidential support further strengthened the interpretation of a heterogeneous evidence base.

The findings should nevertheless be interpreted with several limitations in mind. Because the primary search was limited to WoS and CNKI, retrieval bias cannot be completely excluded despite the supplementary sensitivity assessment. The review was not prospectively registered and no formal written protocol was established. Much of the evidence was cross-sectional or observational, while intervention studies varied in design and methodological strength. Evidence was also concentrated in urban and eastern settings, and direct contributions from people living with dementia were identified in only nine reports.

Future research should focus on the gaps exposed by the synthesis, particularly those that remain weakly examined at cross-setting interfaces. Financing and eligibility require particular attention: studies should examine how eligibility rules, payment arrangements, funded caregiver support, and provider incentives operate across care settings and whether they facilitate or obstruct continuity. Cross-level research should prospectively evaluate referral and transition processes, transferable care information, coordination responsibility, and accountability arrangements, including their effectiveness, implementation requirements, and costs. Caregiver-support and workforce interventions also need comparative and longer-term evaluation under routine service conditions, especially in rural and central and western China. Greater direct involvement of people living with dementia is needed to ensure that future service and governance models reflect their own priorities as well as those of caregivers and providers.

## 5 Conclusion

Family, community, and institutional services for people living with dementia coexist in mainland China; reports directly examining interfaces describe weaknesses in the connections among them. The directly documented fragmentation mainly concerned referral and transition, information continuity, and the transfer or sharing of responsibility, while financing and eligibility interfaces remain comparatively underexamined. Available cross-setting evidence on integration concerned continued support across settings, clearer coordination roles, referral and feedback, and multidisciplinary or community-linked collaboration. The synthesis identifies these cross-setting connections as priorities for further integration efforts, while specific financing, eligibility and accountability arrangements require further evaluation. The policy challenge is not simply to add services, but to make existing and new services work together.

## Data Availability

The original contributions presented in the study are included in the article/Supplementary material, further inquiries can be directed to the corresponding author.
